# Reviewer acknowledgement 2014

**DOI:** 10.1186/s13633-015-0006-2

**Published:** 2015-02-28

**Authors:** Scott Rivkees

**Affiliations:** University of Florida, Gainesville, FL USA

## Abstract

The Editors of the *International Journal of Pediatric Endocrinology* would like to thank all of our reviewers, both external and Editorial Board Members, who have contributed to the journal in Volume 2014 (2014) and whose valuable support is fundamental to the success of the journal.

**Deanna Adkins**

United States of America

**Craig Alter**

United States of America

**Jesus Argente**

Spain

**Richard Auchus**

United States of America

**Ahila Ayyavoo**

India

**Bert Bachrach**

United States of America

**Andrew Bauer**

United States of America

**Giorgio Bedogni**

Italy

**Sheri Berenbaum**

Afghanistan

**Petter Bjornstad**

United States of America

**Gianni Bona**

Italy

**Walter Bonfig**

Germany

**Stuart Brink**

United States of America

**Raúl Calzada**

Mexico

**Dionisios Chrysis**

Greece

**Louise Cominato**

Brazil

**Mehul Dattani**

United Kingdom

**Catherine Dinauer**

United States of America

**Elizabeth Estrada**

United States of America

**Erica Eugster**

United States of America

**Amy Fleischman**

United States of America

**Gary Francis**

United States of America

**Michael Freemark**

United States of America

**Heather Gilbertson**

Australia

**Matti Hero**

Finland

**Khalid Hussain**

United Kingdom

**Jan Idkowiak**

United Kingdom

**Craig Jefferies**

New Zealand

**George Jeha**

United States of America

**Alexander Jorge**

Brazil

**Nicholas Jospe**

United States of America

**Chirag Kapadia**

United States of America

**Paul Kaplowitz**

United States of America

**Cengiz Kara**

Turkey

**Lefkothea Karaviti**

United States of America

**Mimi Kim**

United States of America

**Stephen La Franchi**

United States of America

**Andrew Lane**

United States of America

**Peter Lee**

United States of America

**Claudio Maffeis**

Italy

**Farid Mahmud**

Canada

**Thais Manna**

Brazil

**Nelly Mauras**

United States of America

**Tom Mazur**

United States of America

**Angelika Mohn**

Italy

**Dick Mul**

Netherlands

**Anna Nordenström**

Sweden

**Jerrold Olshan**

United States of America

**Jennifer Osipoff**

United States of America

**Kenneth Robertson**

United Kingdom

**Maria Belen Roldan Martin**

Spain

**Arlan Rosenbloom**

United States of America

**Judith Ross**

United States of America

**Paul Saenger**

United States of America

**David Sandberg**

United States of America

**Dorothy Shulman**

United States of America

**Peter Simm**

Australia

**Jill Simmons**

United States of America

**Phyllis Speiser**

United States of America

**Dennis Styne**

United States of America

**Surendra Varma**

United States of America

**Maria Vogiatzi**

United States of America

**Emily Walvoord**

United States of America

**Benjamin Wheeler**

New Zealand

**Amy Wisniewski**

United States of America

**Jan-Maarten Wit**

Netherlands

**Joseph Wolfsdorf**

United States of America

**Stefan Wudy**

Germany

**Tohru Yorifuji**

Japan

